# Prevalence and incidence of genital warts and cervical Human Papillomavirus infections in Nigerian women

**DOI:** 10.1186/s12879-018-3582-y

**Published:** 2019-01-07

**Authors:** Eileen O. Dareng, Sally N. Adebamowo, Ayotunde Famooto, Oluwatoyosi Olawande, Michael K. Odutola, Yinka Olaniyan, Richard A. Offiong, Paul P. Pharoah, Clement A. Adebamowo

**Affiliations:** 1 Department of Public Health and Primary Care, University of Cambridge, Cambridge, UK; 2 Institute of Human Virology Nigeria, Abuja, Nigeria; 3 Greenebaum Comprehensive Cancer Center, University of Maryland School of Medicine, Baltimore, MD USA; 4 Department of Epidemiology and Public Health, University of Maryland School of Medicine, 725 W. Lombard St. Suite 445, Baltimore, MD 21201 USA; 5 Department of Obstetrics and Gynecology, National Hospital, Abuja, Nigeria; 6 Department of Obstetrics and Gynecology, University of Abuja Teaching Hospital, Abuja, Nigeria; 7 Institute of Human Virology, University of Maryland School of Medicine, Baltimore, MD USA

**Keywords:** Genital warts, Epidemiology, HPV, HIV, Genital tract infection

## Abstract

**Background:**

Genital warts are important causes of morbidity and their prevalence and incidence can be used to evaluate the impact of HPV vaccination in a population.

**Methods:**

We enrolled 1020 women in a prospective cohort study in Nigeria and followed them for a mean (SD) of 9 (4) months. Nurses conducted pelvic examinations and collected ectocervical samples for HPV testing. We used exact logistic regression models to identify risk factors for genital warts.

**Results:**

The mean age of study participants was 38 years, 56% (535/962) were HIV-negative and 44% (427/962) were HIV-positive. Prevalence of genital warts at enrolment was 1% (4/535) among HIV-negative women, and 5% (23/427) among HIV-positive women. Of 614 women (307 HIV negative and 307 HIV positive women) for whom we could compute genital wart incidence, it was 515 (95% CI:13–2872) per 100,000 person-years in HIV-negative and 1370 (95% CI:283–4033) per 100,000 person-years in HIV-positive women. HIV was associated with higher risk of prevalent genital warts (OR:7.14, 95% CI:2.41–28.7, *p* < 0.001) while higher number of sex partners in the past year was associated with increased risk of incident genital warts (OR:2.86, 95% CI:1.04–6.47. *p* = 0.04). HPV11 was the only HPV associated with prevalent genital warts in this population (OR:8.21, 95% CI:2.47–27.3, *p* = 0.001).

**Conclusion:**

Genital warts are common in Nigeria and our results provide important parameters for monitoring the impact of future HPV vaccination programs in the country. HIV infection and number of sexual partners in past year were important risk factors for prevalent and incident genital warts respectively.

## Introduction

Genital warts are common manifestations of genital Human Papillomavirus (HPV) infections [1]. They are commonly associated with HPV6 and HPV11 [2]. However, many other HPV types have also been isolated in genital warts, including HPV 2, 40, 42, 43 and 54 [3, 4]. Although there is rising prevalence and incidence of genital warts in the general population in Europe and the United States, countries such as Australia and England with high HPV vaccination coverage have reported a decline in the burden of genital warts among young women [1, 5–8]. In contrast, little is known about genital warts in Sub-Saharan Africa (SSA) [9]. Most studies on genital warts in SSA have been conducted in high risk populations such as people attending sexually transmitted diseases’ (STD) clinics or commercial sex workers [9]. Other studies were conducted among women receiving antenatal care [9].

Genital warts can present with four morphologic features – condylomata acuminata, smooth or flat papular lesions, or keratotic warts. Apart from their appearance, they are often symptomless except in some cases of vulvar warts which may cause dyspareunia and discomfort; penile warts and pruritus; vaginal warts and vaginal discharge, bleeding, obstruction of the birth canal, and neonatal infection which may lead to juvenile onset recurrent papillomatosis; perianal and intra-anal warts that cause pain, bleeding on defecation and pruritus [10–14]. Genital warts can have profound effects on patients’ quality of life [15]. They may regress spontaneously or remain quiescent for extended periods. They can be refractory to treatment with high recurrence rates leading to substantial health care costs [16]. In 2004, the estimated economic burden of genital warts exceeded $220 million in the US, [17] and $64 million in Spain [18]. Data on economic costs are not available for SSA countries.

Studies show that vaccination with the quadrivalent and nonavalent HPV vaccine, which offers protection against HPV 6 and 11, can reduce the incidence of genital warts [19]. This has renewed interest in estimating and monitoring the burden of anogenital warts as an early indicator of the impact of HPV vaccination programs. In Nigeria, there are isolated, small-sized, pilot HPV vaccination projects but no systematic, national or regional programs [20]. Therefore, estimating the pre-vaccination burden of genital warts would be of practical utility for future monitoring and evaluation projects.

In this study, we describe the prevalence, incidence, associated risk factors and cervical HPV types associated with genital warts in Nigerian women.

## Methods

### Study population

We conducted cervical cancer awareness meetings in town halls, HIV outpatient clinics, general outpatient and gynaecology outpatient clinics in two hospitals (National Hospital, Abuja and University of Abuja Teaching Hospital) in Abuja, Nigeria. We invited 1300 women to participate in cervical cancer screening programs at these two hospitals and enrolled 1020 (78%) women into a prospective cohort to investigate the prevalence and incidence, host and viral determinants of persistent HPV infection in HIV negative and HIV positive women in Nigeria between 2012 and 2013. This study population has been previously described [21, 22]. We included women over 18 years old with prior history of penetrative vaginal intercourse and excluded women who were pregnant, had previous hysterectomies, had previous history of cervical cancer or its precursor lesions or were unable to attend follow up visits.

Trained nurses administered questionnaires to collect information on participants’ sociodemographic and lifestyle characteristics. Nine nurses (five at the National Hospital Abuja and four at the University of Abuja Teaching Hospital) performed physical and gynecologic examinations on participants and collected ecto-cervical cell scrapings for HPV determination. All nurses had at least two years of experience in cervical cancer screening, with at least a Bachelor’s degree in nursing. All participants were scheduled to return six months after enrollment, when the baseline procedures were repeated. Participants were compensated for travel costs and time. We ascertained HIV status from participant’s self report of a previous test and those without a history of HIV testing were referred to a counselling and testing center.

### HPV DNA detection

We used the SPF_10_LiPA_25_ system version 1 for HPV DNA detection [23]. The LiPA assay can identify 25 high-risk and low-risk HPV types. Specimens that were positive for HPV DNA, but did not hybridize with any of the probes in LiPA_25_ were labeled as HPV undetermined (HPV U).

### Genital warts identification

Nurses identified the presence of genital warts by direct visual inspection with bright light and magnification. We used the ICD-10-CM Diagnosis Code A63.0 to define genital warts and included lesions on the labia, vagina, cervix, outer pubic and anal areas. Prevalent cases were identified at enrolment. Incident cases were identified at follow-up in women without any visible genital warts at enrolment and no prior history of self-reported genital warts.

### Statistical analysis

We compared the baseline characteristics of HIV positive and HIV negative women using *t*-tests for continuous variables and Fisher’s exact tests for categorical variables. We used exact logistic regression models to investigate potential risk factors associated with prevalent and incident genital warts. Potential risk factors evaluated included sociodemographic (age, education, socioeconomic status, marital status); sexual and vaginal practices (HIV status, condom use, sexual debut age, lifetime number of partners, number of partners in past year, douching practice) and body mass index. All variables were updated at the six month visit and the updated data was used for incident warts analysis. All risk factors with *p ≤* 0.20 in age adjusted models were included in multivariable models. We used HIV and age adjusted Poisson regression models, with each HPV type modelled as a binary variable, to evaluate the association between each HPV type and prevalent genital warts.

To minimize bias in incidence estimates, we used multiple imputation methods to impute the missing information on person time at risk using STATA *mi impute regress* module [24]. All analysis of incident genital warts was based on the imputed data were conducted using Stata version 15 (StataCorp LP, College Station, Texas).

### Ethical approval

Ethical approval was obtained from the National Health Research Ethics Committee of Nigeria (NHREC/01/01/2007–01/08/2016) and the University of Maryland Institutional Review Board (HCR-HP-00051495-2). All participants provided written informed consent.

## Results

Of the 1020 women enrolled, 58 were excluded due to missing HIV and HPV data (41 missing HIV results, 13 missing baseline HPV results, 4 missing both HIV and HPV) results leaving 962 women available for prevalent genital warts analyses. Most participants (72%, 692/962) completed follow-up visits. Of these 692 women, we excluded 62 participants whose HPV results were missing at follow-up leaving 630 women for genital wart incidence analysis. Sixteen of these women had prevalent genital warts at the enrolment visit. Therefore, they were no longer at risk for incident genital warts and were subsequently excluded leaving 614 women. Among these 614 women, we imputed person time at risk for 4% (22/614) of participants who were missing follow-up visit date. All instances of missing data were in women without incident genital warts. Sensitivity analysis, comparing results from models with and without imputed data did not show any significant differences. We present results from models with the imputed data in order to reduce loss of information that would arise from listwise deletion of cases.

### Characteristics of study population

Almost half of the women (44%, 427/962) were HIV positive (Table 1). Mean age (SD) at enrolment was 38 (8) years and at sexual debut was 20 (4) years. Most participants obtained more than six years of formal education (89%, 858/962) and were married (67%, 647/962). Prevalence of smoking (1%, 13/962) and use of oral contraceptives (10%, 93/962) were low. Only a third (33%, 303/962) of the women were neither overweight nor obese. Mean (SD) follow-up time was 9 (4) months. None of the participants had been previously vaccinated for HPV or received treatment for genital warts.

**Table 1:** Characteristics of study participants by HIV status at enrolment

	Total (*N* = 962)	HIV negative (*N* = 535)	HIV positive (*N* = 427)	*p*-value
Sociodemographic characteristics				
Age, years (Mean, SD)	37.9 (7.72)	38.4 (7.90)	37.2 (7.42)	0.02
Nature of residence (N, %)				< 0.001
Urban	431 (45.0)	283 (53.1)	148 (34.9)	
Semirural	367 (38.4)	180 (33.8)	187 (44.1)	
Rural	159 (16.6)	70 (13.1)	89 (30.0)	
Socioeconomic status (N, %)				< 0.001
Low	375 (39.0)	152 (28.4)	223 (52.2)	
Middle	384 (39.9)	226 (42.2)	158 (37.0)	
High	203 (21.1)	157 (29.4)	46 (10.8)	
Education (N, %)				< 0.001
≤ 6 years	104 (10.8)	45 (8.40)	59 (13.8)	
7–12 years	602 (62.7)	294 (55.1)	308 (72.3)	
> 12 years	254 (26.5)	195 (36.5)	59 (13.9)	
Marital status (N, %)				< 0.001
Married	647 (67.3)	421 (78.7)	226 (52.9)	
Not married	315 (32.7)	114 (21.3)	201 (47.1)	
Lifestyle characteristics				
Age at sexual debut, years (Mean, SD)	20.1 (4.04)	20.8 (4.24)	19.2 (3.59)	< 0.001
Lifetime number of partners (Mean, SD)	3.43 (3.34)	2.85 (2.35)	4.16 (4.16)	< 0.001
Number of partners in preceding year (Mean, SD)	1.00 (0.47)	1.00 (0.34)	1.00 (0.60)	0.86
Ever smoked^a^ (N, %)	13 (1.35)	4 (0.75)	9 (2.11)	0.09
Alcohol Consumption^b^ (N, %)	129 (13.5)	70 (13.2)	59 (13.9)	0.78
Ever douche (N, %)	606 (63.1)	319 (59.6)	287 (67.4)	0.02
Regularly douche in last 3 months (N, %)	544 (56.5)	284 (53.1)	260 (60.9)	0.32
Condom use (N, %)	290 (30.2)	102 (19.1)	188 (44.0)	< 0.001
Oral contraceptive use (N, %)	93 (9.67)	38 (8.90)	55 (10.3)	0.51
Clinical characteristics				
Body Mass Index, kg/m^2^ (N, %)				< 0.001
Normal weight, 18.5–24.9	303 (33.4)	136 (26.6)	167 (42.0)	
Overweight, 25.0–29.9	324 (35.6)	184 (35.9)	140 (35.3)	
Obese, ≥ 30.0	282 (31.0)	192 (37.5)	90 (22.7)	
Vaginal pH (N, %)				0.21
< 4.5	73 (7.59)	47 (8.79)	26 (6.09)	
4.5–5.5	68 (7.07)	34 (6.36)	34 (7.96)	
> 5.5	821 (85.3)	454 (84.9)	367 (85.9)	
Diagnosis of vaginal infection in last 3 months^c^ (N, %)	23 (2.39)	17 (3.18)	6 (1.41)	0.09
Currently on antiretroviral therapy (N, %)	–	–	395 (92.5)	

a Ever smoked up to 100 cigarrette sticks in lifetime

b Consumption of at least one unit of alcohol every three months

c Infections include bacterial vaginosis, Candidiasis, Trichomonas, Herpes, Chlamydia, Pelvic inflammatory disease, gonorrhoea, syphilis and genital warts

There was no significant difference among the HIV negative and HIV positive women with respect to the number of sexual partners in past year, smoking history, alcohol consumption, vaginal pH, and previous history of vaginal infections. More HIV positive women resided in rural environments, had low socioeconomic status, were less educated, more likely to be widowed and more likely to report condom use compared to HIV negative women (Table 1). The distribution of low-risk and high-risk HPV types in this population has been previously described [22].

### Factors associated with prevalent and incident genital warts

In univariate analysis, age, socioeconomic status, HIV status, condom use and BMI were associated with prevalent genital warts, while number of sex partners in the past year was associated with incident genital warts (Table 2). In a multivariable model adjusted for age, HIV infection was associated with a higher risk of prevalent genital warts (OR: 7.14, 95% CI: 2.41–28.7. *p* < 0.0001) while higher number of sex partners in the past year was associated with an increased risk of incident genital warts (OR: 2.86, 95% CI: 1.04–6.47. *p* = 0.04).

**Table 2:** Association between participant sociodemographic, potential risk factors and genital warts

Variable	Prevalent genital warts				Incident genital warts			
	Total N = 962	*n* (%)	Odds Ratio (95% CI)	*p*	Total *N* = 614	*n* (%)	Odds Ratio (95% CI)	*p*
Age			0.94 (0.88–0.99)	0.01			0.92 (0.80–1.06)	0.28
Education, years completed								
≤ 6 years	104	5 (5)	Reference (1.00)		70	0 (0)	Reference (1.00)	
7–12	602	15 (3)	0.51 (0.17–1.82)	0.32	382	3 (1)	0.73 (0.08 – Inf)	1.00
> 12	254	7 (3)	0.56 (0.15–2.30)	0.50	160	1 (1)	0.45 (0.01 – Inf)	1.00
Socio-economic status								
Low	375	15 (4)	Reference (1.00)		238	3 (1)	Reference (1.00)	
Middle	384	11 (3)	0.71 (0.30–1.68)	0.51	251	1 (0)	0.32 (0.01–3.98)	0.59
High	203	1 (0)	0.12 (0.00–0.78)	0.02	125	0 (0)	0.51 (0.00–4.78)	0.58
Marital status								
Married	647	15 (2)	Reference (1.00)		405	2 (0)	Reference (1.00)	
Not married	315	12 (4)	1.67 (0.70–3.87)	0.27	209	2 (1)	1.95 (0.14–27.1)	0.84
Douche								
No	355	8 (2)	Reference (1.00)		218	1 (0)	Reference (1.00)	
Yes	606	19 (3)	1.40 (0.58–3.74)	0.56	395	3 (1)	1.64 (0.13–86.5)	1.00
HIV status								
No	535	4 (1)	Reference (1.00)		307	1 (0)	Reference (1.00)	
Yes	427	23(5)	7.54 (2.54–30.2)	< 0.001	307	3 (1)	2.90 (0.23–153)	0.65
Condom use								
No	672	13 (2)	Reference (1.00)		433	2 (0)	Reference (1.00)	
Yes	290	14 (5)	2. 57 (1.10–6.02)	0.03	181	2 (1)	2.32 (0.17–32.3)	0.70
Age at sexual initiation			0.97 (0.88–1.07)	0.56			0.87 (0.64–1.15)	0.39
Per partner increase in total lifetime sex partners			1.05 (0.96–1.13)	0.23			1.04 (0.77–1.20)	0.52
Per partner increase in total sex partners in past year			1.18 (0.49–2.37)	0.75			3.16 (1.20–6.99)	0.02
Body Mass Index, Kg/m^2^								
Normal weight	303	12 (4)	Reference (1.00)		187	1 (1)	Reference (1.00)	
Overweight	324	6 (2)	0.46 (0.14–1.34)	0.18	214	1 (0)	0.90 (0.01–70.9)	1.00
Obese, ≥ 30	282	7 (2)	0.62 (0.20–1.73)	0.44	178	1 (1)	1.07 (0.01–84.1)	1.00

**Fig. 1:**
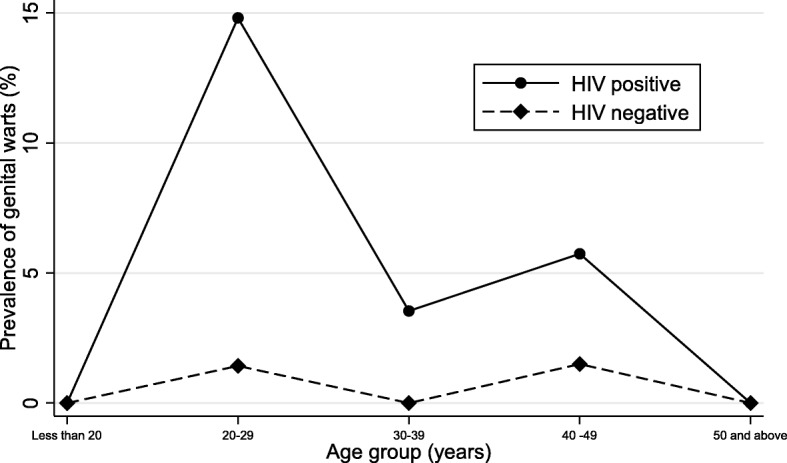
Point prevalence of genital warts at enrolment by HIV status and age

**Fig. 2:**
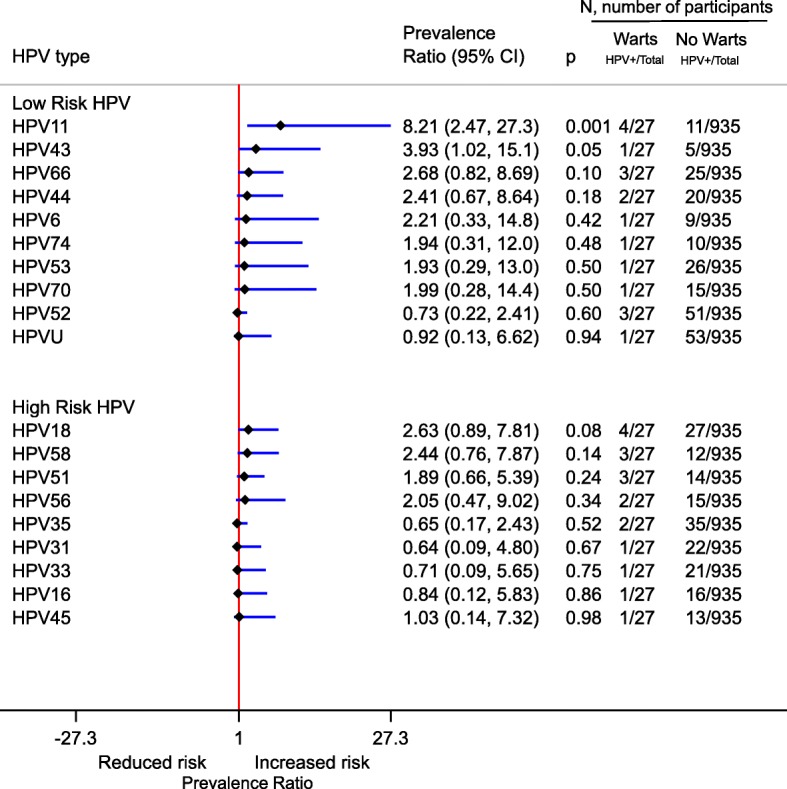
Association between cervical type-specific low-risk and high-risk HPV and prevalent genital warts, adjusted for age and HIV status

Prevalence of genital warts was low below 20 years but rose rapidly to peak at 20–29 years followed by a decline till about 40 years when there was a second but smaller peak (Fig. 1). In a multivariable model adjusted for HIV and age, HPV11 was the only HPV type significantly associated with prevalent genital warts (OR: 8.21, 95% CI: 2.47–27.3. *p* = 0.001) (Fig. 2). HPV6 was not significantly associated with prevalent genital warts (OR: 2.21, 95% CI: 0.33–14.8. *p* = 0.42). Due to the reported association between HPV6 and genital warts, we included both HPV6 and HPV11 in a multivariable model adjusted for age and HIV. The results remained largely unchanged for both HPV11 and HPV6.

### Genital warts in HIV negative women

Of the 962 women enrolled, 535 were HIV negative. Among these HIV negative women, the prevalence of genital warts at enrolment was 1% (4/535). Two of these women (50%, 2/4) were positive for any HPV. The HPV types detected were HPV11 (25%, 1/4) and HPV18 (25%, 1/4) (Table 3). Some 57% (307/535) of these women returned for follow-up and had complete data for incident genital warts analysis. Only one of them developed incident genital warts during the follow-up period. The total person time at risk for genital warts for HIV negative women was 194 person-years, resulting in an incidence rate of 515 cases (95% CI: 13–2872) per 100,000 person-years. Cervical HPV was not detected in this woman, either at enrolment or during follow-up eight months later.

**Table 3:** Cervical HPV type distribution in 27 women with prevalent genital warts at enrolment by HIV status

Cervical HPV type	Prevalent genital warts		
	Total (*n* = 27) *	HIV- (n = 4)	HIV + (*n* = 23)
	Number infected (%)	Number infected (%)	Number infected (%)
Low risk HPV			
HPV 6	1 (4)	0 (0)	1 (4)
HPV 11	4 (15)	1 (25)	3 (13)
HPV 43	1(4)	0 (0)	1 (4)
HPV 44	2 (7)	0 (0)	2 (9)
HPV 53	1 (4)	0 (0)	1 (4)
HPV 66	3 (11)	0 (0)	3 (13)
HPV 70	1 (4)	0 (0)	1 (4)
HPV 74	1 (4)	0 (0)	1 (4)
HPV U	1 (4)	0 (0)	1 (4)
High risk HPV			
HPV 16	1 (4)	0 (0)	1 (4)
HPV 18	4 (15)	1 (25)	3 (13)
HPV 31	1 (4)	0 (0)	1 (4)
HPV 33	1 (4)	0 (0)	1 (4)
HPV 35	2 (7)	0 (0)	2 (9)
HPV 45	1 (4)	0 (0)	1 (4)
HPV 51	3 (11)	0 (0)	3 (13)
HPV 52	3 (11)	0 (0)	3 (13)
HPV 56	2 (7)	0 (0)	2 (9)
HPV 58	3 (11)	0 (0)	3 (13)
Any HPV infection	23 (85)	2 (50)	21 (91)
Single HPV infection	15 (56)	2 (50)	13 (57)
Multiple HPV infection			
2 types	3 (11)	0 (0)	3 (13)
3 types	1 (4)	0 (0)	1 (4)
4 types	3 (11)	0 (0)	3 (13)
5 types	1 (4)	0 (0)	1 (4)

*Numbers add up to > 27 because some women (8/27) had multiple infections ranging from two to five HPV types

### Genital warts in HIV positive women

Of the 962 women enrolled, 427 were HIV positive. Prevalence of genital warts among these HIV positive women at enrolment was 5% (23/427). Among those with prevalent genital warts, 57% (13/23) had single HPV infections, 35% (8/23) had multiple HPV infections ranging from two to five types and 8% (2/23) did not have any HPV infections (Table 3). The most prevalent low-risk HPV types detected in these HIV positive women with genital warts were HPV11 (13%, 3/23) and HPV66 (13%, 3/23). While, the most prevalent high-risk types were HPV18 (13%, 3/23), HPV51(13%, 3/23), HPV52 (13%, 3/23) and HPV58 (13%, 3/23) (Table 3).

Some 72% (307/427) of HIV positive women returned for follow-up. There were three incident cases of genital warts in these women during 219 person-years of follow-up, resulting in an incidence rate of 1370 cases (95% CI: 283–4033) per 100,000 person-years. The HPV types detected at follow-up in these three women were HPV 35 and HPV 52 in one woman, and none in the other two women. At enrolment, all three women had multiple HPV types. There were three HPV types in two of the women (HPV 11, HPV 54, HPV 68 in one woman and HPV 35, HPV 52, HPV 54 in the second woman) and four HPV types (HPV 44, HPV 53, HPV 58, HPV 66) in one of the women.

## Discussion

We identified HIV infection as a risk factor for prevalent genital warts and higher number of sex partners in the past year as a risk factor for incident genital warts in this study. Cervical HPV11, but not HPV6 infection was associated with prevalent genital warts.

Previous studies have shown that HIV positive women are more likely to be infected with HPV, have multiple HPV infections, [25] and have slower clearance compared with HIV negative women [26]. Clearance of HPV infection is mediated by a cell-mediated immune response, and the cellular response against genital warts includes an antigen specific CD4+ Th1 response [27]. Therefore, the increased risk of genital warts in HIV positive women is likely due to the reduced ability of the immune system to control HPV infections, as well the shared risk factors for transmission of HIV and HPV infections.

Given the ubiquity of HPV infections, the number of sexual partners in the past year is a corollary of exposure to new HPV infections from new sexual partners. Women with a higher number of sexual partners in the preceding year are more likely to have been exposed to new HPV infections from new sexual partners than women who report fewer number of sexual partners [28]. Genital warts have a short incubation period, with about 65% of people exposed to partners with genital warts, developing lesions within 1–8 months [29]. Therefore, the three-fold increased risk of incident genital warts with each additional new partner in the preceding year observed in our study likely reflects the clinical manifestations of newly acquired HPV infections.

Prevalence of HPV6 was 4% (1/27) in women with genital warts compared to the overall prevalence of 1% (10/962) in the study population, while prevalence of HPV11 was 15% (4/27) in women with genital warts compared to the overall prevalence of 2% (15/962) in the study population [22]. In age adjusted models, HPV11, but not HPV6, was significantly associated with prevalent genital warts. The prevalence of cervical HPV6 infection among women with prevalent genital warts in our study are lower than that from other studies in Burkina Faso (6%) [30], South Africa (5%) [30], Germany (67%) [31], United States (17%) [4], and England (33%) [32]. These differences may reflect the relative contributions of non-HPV 6/11 infections to the burden of genital warts, and differences in the age distribution of the study populations. Due to the recurring nature of genital wart infections and the transient nature of most cervical HPV infections, inferences based on HPV detection at time of genital wart diagnosis need to be interpreted with caution. Intermittent detection of persistent HPV infection may be due to episodic fluctuation in viral load levels including below the detection limit of many HPV DNA assays [33]. HPV infections may also go through a phase of latency in the basal cells of the cervical epithelium during which period the virus may be undetectable [3].

The prevalence of genital warts among HIV negative women in our study is lower than that found in HIV negative, pregnant women in Malawi where prevalence was approximately 2% [9]. This is consistent with results from previous studies which show that HPV prevalence tend to be higher among pregnant compared to age-matched non pregnant women as a result of temporary immunosuppression and increased steroid hormones during pregnancy [34]. The prevalence of genital warts in HIV positive women in our study is similar to that in a recent study of HIV positive women in South Africa (6%) but slightly lower than that in Burkina Faso (8%) [30]. Geographic variations in any HPV prevalence, cultural environment, sexual behavior, HPV viral characteristics and host susceptibility may explain these findings. In a meta-analysis assessing the burden of any HPV infection in women with normal cytological findings, standardized by country specific population sizes, any HPV prevalence was higher in Eastern Africa (34%) compared to Western Africa, which includes Nigeria (20%) [35]. Similarly, the incidence and prevalence of other HPV associated diseases such as cervical cancer is higher in Eastern Africa compared to Western Africa [35].

We observed a peak prevalence of genital warts in women aged 20–29 years followed by a decline till about 40–49 years old where there was a second but smaller peak. This result is consistent with our previous findings on prevalent low risk HPV infection in this study population and the epidemiology of several other STIs from other populations where the highest peaks are observed in young people soon after sexual debut, with a secondary peak in middle age in some populations [22, 36]. Possible explanations for the bimodal pattern include the reactivation of latent HPV infections in later life due to waning of immunity, acquisition of new HPV infections due to new sexual partners and possibly cohort effects [37].

The incidence rates of 515 in HIV negative and 1370 per 100,000 person-years in HIV positive women in our study are higher than that from European and North American studies [5]. In a systematic review of population based incidence rates using data from medical chart reviews and retrospective administrative databases in European and North American populations, Patel et al. reported incidence rates of genital warts per 100,000 person-years ranging from 76 in Germany to 430 in Italy [5]. Incidence of genital warts based on clinical pelvic examinations tend to be higher than that obtained from medical chart reviews or administrative databases because patients may be unwilling or unable to present to health care facilities [5]. Additionally, medical records and administrative databases may be incomplete and the quality of information provided by different health care workers may vary significantly.

Few studies of the incidence of genital warts have been conducted in SSA and these have been in high risk populations such as HIV negative commercial sex workers in Burkina Faso where the incidence was 1100 per 100,000 person-years, [9] and in women younger than 40 years, attending an STD clinic in Nigeria with incidence of 2700 per 100,000 person-years [9]. A recent study found genital wart incident rates of 2470 and 2330 per 100,000 person years in HIV positive women in Burkina Faso and South Africa respectively [30]. These rates are higher than what we observed in HIV positive women in our study. Factors related to differences between the study populations and  differences in HIV epidemiology may account for these findings.

We found that women younger than 30 years old or HIV negative were more likely to be lost to follow up in our study. Considering that the prevalence of genital warts was higher in women less than 30 years old in this study, it is possible we underestimated the incidence of genital warts as a result. As incidence estimates were stratified by HIV status, the increased likelihood of being lost to follow up among HIV negative women, is more likely to influence estimates for HIV negative but not HIV positive women. We previously published a detailed study of predictors of attrition in this cohort and identified age and HIV status as important risk factors [38].

We found that HIV positive women were significantly more likely to report condom use than HIV negative women. This finding may reflect recent efforts by the President’s Emergency Plan for AIDS Relief (PEPFAR) program to promote condom use among sexually active HIV positive individuals to reduce risk of HIV and other sexually transmitted infections (STI) [39]. It is plausible that there are differences in the psychological and recall processes for self reported sexual history among HIV negative and HIV positive women. HIV positive women may feel a sense of personal responsibility in preventing the transmission of HIV and other STIs to sexual partners and therefore may over report condom use, which they may perceive as a more socially desirable response. This is supported by our previous study showing less reliability in reporting sexual behaviour among HIV positive compared to HIV negative women in this study population [21].

To our knowledge, this is the first longitudinal study to describe the prevalence and incidence of genital warts among HIV negative and HIV positive women who are neither pregnant nor considered to be at increased risk for other STDs in West Africa. Due to the recurring nature of genital warts, newly detected cases of genital warts may truly be incident cases or may be recurrent episodes. In our study, we collected self-reported history of previous genital warts and excluded these cases from incidence analyses. However, it is possible that there may be some residual confounding as a result of inaccuracies in the self-reported history.

HPV detection was based on cells obtained from the cervix which may not be representative of the causative HPV types in the genital warts. Previous studies have reported varying degrees of type specific concordance between cervical HPV and HPV isolated from genital warts [4, 31]. In these studies, agreements for HPV6 detected in genital swabs and cervical swabs among young women with genital warts ranged from 16% in a US study, [4] to 71% in a German study [31]. Therefore, it is likely that the prevalence estimates of HPV types in our study underestimate the true prevalence of the different HPV types in genital warts. Despite this limitation, our results provide some information on the HPV type distribution among Nigerian women with genital warts. Further studies, that directly sample gential warts are needed to better understand genital warts epidemiology in Nigeria.

Other limitations of our study include small sample size, the small number of incident genital warts despite the relatively large follow-up person time and the absence of HIV parameters such as viral load and CD4+ count. Therefore, our effect estimates for incident genital warts may be unstable. In our study design, we purposively enroled HIV negative and HIV positive women. Therefore, the prevalence of HIV in our study population does not represent HIV prevalence in the Nigerian female population. Hence, we provide prevalence and incidence estimates separately for HIV negative and HIV positive populations.

Genital warts diagnosis in our study was based on visual identification with bright light. Without histological confirmation, it is possible that some other genital conditions such as seborrheic keratoses, dysplastic and benign nevi, molluscum contagiosum and neoplastic lesions may have been misclassified as genital warts [13]. However, biopsy is not routinely recommended for genital warts diagnosis, and the nurses in our study were trained to rule out these other genital conditions and refer lesions suspicious of neoplasia.

## Conclusion

Our study provides important information on the epidemiology of genital warts among women in Nigeria. The results can be used for monitoring and modelling effectiveness of the public health impact of future HPV vaccination programs in Nigeria. Our findings of a high burden of genital warts and the association between cervical HPV11 and genital warts support the use of either the quadrivalent or nonavalent vaccine for genital wart prevention in future HPV vaccination programs in Nigeria.
